# *PIK3CA* Activating Mutation in Colorectal Carcinoma: Associations with Molecular Features and Survival

**DOI:** 10.1371/journal.pone.0065479

**Published:** 2013-06-13

**Authors:** Christophe Rosty, Joanne P. Young, Michael D. Walsh, Mark Clendenning, Kristy Sanderson, Rhiannon J. Walters, Susan Parry, Mark A. Jenkins, Aung Ko Win, Melissa C. Southey, John L. Hopper, Graham G. Giles, Elizabeth J. Williamson, Dallas R. English, Daniel D. Buchanan

**Affiliations:** 1 Cancer and Population Studies Group, Queensland Institute of Medical Research, Herston, Australia; 2 University of Queensland, School of Medicine, Herston, Australia; 3 Envoi Pathology, Herston, Australia; 4 Sullivan and Nicolaides Pathology, Taringa, Australia; 5 Department of Gastroenterology and Hepatology, Middlemore Hospital, Auckland, New Zealand; 6 New Zealand Familial Gastrointestinal Cancer Service, Auckland Hospital, Auckland, New Zealand; 7 Centre for Molecular, Environmental, Genetic and Analytic Epidemiology, The University of Melbourne, Parkville, Australia; 8 Genetic Epidemiology Laboratory, Department of Pathology, The University of Melbourne, Parkville, Australia; 9 Cancer Epidemiology Centre, Cancer Council Victoria, Carlton, Australia; Howard University, United States of America

## Abstract

Mutations in *PIK3CA* are present in 10 to 15% of colorectal carcinomas. We aimed to examine how *PIK3CA* mutations relate to other molecular alterations in colorectal carcinoma, to pathologic phenotype and survival. *PIK3CA* mutation testing was carried out using direct sequencing on 757 incident tumors from the Melbourne Collaborative Cohort Study. The status of O-6-methylguanine-DNA methyltransferase (MGMT) was assessed using both immunohistochemistry and methyLight techniques. Microsatellite instability, CpG island phenotype (CIMP), *KRAS* and *BRAF* V600E mutation status, and pathology review features were derived from previous reports. *PIK3CA* mutation was observed in 105 of 757 (14%) of carcinomas, characterized by location in the proximal colon (54% vs. 34%; *P*<0.001) and an increased frequency of *KRAS* mutation (48% vs. 25%; *P*<0.001). High-levels of CIMP were more frequently found in *PIK3CA*-mutated tumors compared with *PIK3CA* wild-type tumors (22% vs. 11%; *P* = 0.004). There was no difference in the prevalence of *BRAF* V600E mutation between these two tumor groups. *PIK3CA*-mutated tumors were associated with loss of MGMT expression (35% vs. 20%; *P* = 0.001) and the presence of tumor mucinous differentiation (54% vs. 32%; *P*<0.001). In patients with wild-type *BRAF* tumors, *PIK3CA* mutation was associated with poor survival (HR 1.51 95% CI 1.04–2.19, *P* = 0.03). In summary, *PIK3CA*-mutated colorectal carcinomas are more likely to develop in the proximal colon, to demonstrate high levels of CIMP, *KRAS* mutation and loss of MGMT expression. *PIK3CA* mutation also contributes to significantly decreased survival for patients with wild-type *BRAF* tumors.

## Introduction

*PIK3CA* (*phosphatidylinositol-3,4-bisphosphonate 3-kinase, catalytic subunit alpha*) encodes for the catalytic p110-alpha subunit of Phosphatidylinositol 3-Kinase (PI3K) alpha, which orchestrates cell responses including cell proliferation, survival, proliferation, migration and morphology [1], [2]. PI3K is a family of proteins capable of phosphorylating phosphaditylinositols in response to activation of growth factors from various ligands such as epidermal growth factor (EGF), insulin, platelet-derived growth factor (PDGF) or vascular endothelial growth factor (VEGF). The PI3K pathway is normally inhibited by tumor suppressor gene *PTEN* [3]. Alterations in PI3K signaling pathway are frequent events in various tumor types either through inactivation of *PTEN* or by amplification or mutation in *PIK3CA*. Activating mutations in *PIK3CA* have been found in neoplasms of the colorectum, stomach, breast, brain, ovaries, liver and lung [4], [5]. These mutations are concentrated in 2 hot spots of the gene: the helical domain in codons 542 and 545 of exon 9 and the kinase domain in codon 1047 of exon 20. A gain of transformative enzymatic function in both cell lines and orthotopic models results from mutation within both regions [6], [7]. For colorectal carcinoma, the prevalence of *PIK3CA* mutation was reported to be between 7% and 32%, with G>A transversions in exon 9 being the most commonly observed configuration [4], [8], [9], [10], [11], [12], [13], [14], [15], [16], [17], [18], [19], [20], [21].

Recent interest arose for *PIK3CA* as a potential predictive marker of personalized therapy for colorectal carcinoma and a target for specific pharmaceutical agents. *PIK3CA* is a downstream effector of Epidermal Growth Factor Receptor (EGFR), a target for monoclonal antibodies such as cetuximab and panitumumab, leading to inhibition of its signaling cascade. The clinical benefit of this targeted therapy is restricted to wild-type *KRAS* colorectal carcinoma [22], [23]. It has been suggested that the deregulation of *PIK3CA*/*PTEN* might also influence the response to EGFR inhibitors in metastatic disease [24], [25]. PI3K represents a potential new target for specific therapeutic inhibitors with promising molecules currently under clinical trials [26]. More recently, *PIK3CA* mutation was associated with longer survival in patients who use aspirin regularly after diagnosis [27].

The study of the molecular pathology of *PIK3CA*-mutated colorectal carcinoma has produced some conflicting results, as displayed in **table 1**, in particular, its association with CpG Island Methylator Phenotype (CIMP) and O-6-methylguanine-DNA methyltransferase (MGMT) status [12], [13], [17], [18], [21], [25], [28], [29], [30]. The relationship between *PIK3CA* mutation and other molecular pathologic features such as microsatellite instability (MSI) has not been explored in detail. It is unclear whether *PIK3CA* mutation defines a clinically and/or biologically relevant subset of tumors as there is significant overlap with *KRAS* and *BRAF* V600E mutation. Clinical studies testing the effect of *PIK3CA* mutation on patient outcomes have produced conflicting results [8], [9], [13], [15], [17], [19], [28], [31], [32], with the majority of studies showing a mild adverse prognostic effect of *PIK3CA* mutation in various subgroups of patient. Our aim was to explore the clinical, molecular and pathologic characteristics of incident *PIK3CA*-mutated colorectal carcinomas from a large prospective cohort study.

**Table 1:** Summary of results from reported studies on *PIK3CA* mutation in colorectal carcinoma and various associations with molecular and pathological characteristics.

Authors	Number of CRC	Detection Method	*PIK3CA* Mutation	*KRAS* Mutation	*BRAF* Mutation	CIMP-H	MSI-H	*MGMT* Methylation	MGMT Expression	Proximal Colon	Mucinous CRC
Iida *et al* [13]	164	Sanger sequencing	12%	NS	NI	NI	NI	NI	NI	NS	NI
Hsieh *et al* [12]	182	HRM	12%	P = 0.007	No overlap	NI	NI	NI	NI	NI	NI
Whitehall *et al* [21]	829	HRM	14%	P<0.001	NS	P<0.001	NS	P = 0.007	NI	NS	NI
Tian *et al* [25]	381	Sanger sequencing	12%	NS	NS	NI	NI	NI	NI	NI	NI
Liao *et al* [27]	1170	Pyrosequencing	16%	P<0.001	NS	NS	NS	NI	NI	P = 0.03	NI
Garrido-Laguna *et al* [30]	238#	Pyrosequencing	15%	P = 0.03	NS	NI	NI	NI	NI	NI	P = 0.04
Ogino *et al* [18]	450##	Pyrosequencing	18%	P<0.001	NS	NS	NS	NI	NI	NS	NI
Nosho *et al* [17]	590	Pyrosequencing	15%	P<0.001	NS	P = 0.03	NS	P = 0.04	P = 0.01	NS	P<0.001
Kato *et al* [28]	158	Sanger sequencing	11%	NS	NI	NI	NI	NI	NI	NI	NI

# metastatic colorectal carcinoma;

## resectable colorectal carcinoma;

CRC: Colorectal carcinoma;

CIMP-H: High levels of CpG island methylator phenotype;

MSI-H: High levels of microsatellite instability;

HRM: High resolution melting;

NS: Not significant (P>0.05);

NI: Not enough information to determine.

## Patients and Methods

### Study Samples

Participants were enrolled in The Melbourne Collaborative Cohort Study, a prospective cohort study of 41,514 people (17,045 males and 24,469 females) recruited between 1990 and 1994. The design and recruitment of this study have been previously described in detail [33]. The study protocol was approved by the Cancer Council Victoria’s Human Research Ethics Committee and the Human Research Ethics Committee of the Queensland Institute of Medical Research under protocol P799. Participants gave written consent for participation and for the investigators to obtain their medical records. Clinical data were collected from medical charts, colonoscopy and pathology reports. Location in the colon was designated as proximal for tumors located in the cecum, ascending colon and transverse colon and as distal in the descending colon, sigmoid colon and rectum.

### Molecular Pathology

All cases underwent standardized histopathology review by a specialist gastrointestinal pathologist and assessed histologically for the following features: histologic type, mucinous differentiation, grade, tumor margin (circumscribed or infiltrative), tumor budding (non-quantitative method), the presence of tumor infiltrating lymphocytes, and the presence of a residual polyp adjacent to the carcinoma. Mucinous differentiation in the tumor was defined by the presence of pools of extracellular mucin containing clusters of carcinomatous cells or individual tumor cells, including signet ring cells. Tumors were classified as mucinous carcinoma when >50% of analyzed tumor demonstrated mucinous differentiation. Histologic grading was performed as follows: conventional carcinomas were classified as low grade if ≥50% gland formation was present and high grade if <50% gland formation was present; mucinous carcinomas were graded according to the MSI phenotype: low grade if MSI-high was present, high grade if MSI-high was absent.

Immunohistochemistry for mismatch repair proteins MLH1, PMS2, MSH2, MSH6, and for MGMT have been described in previous reports [34], [35]. Tumors showing complete loss of expression compared with the normal colonic mucosa were interpreted as having altered expression of MGMT.

High levels of CpG Island methylator phenotype (CIMP) were defined by methylation of ≥3 of the following markers *RUNX3*, *SOCS1*, *NEUROG1*, *CACNA1G*, and *IGF2. MLH1* gene promoter methylation, *BRAF* V600E mutation analysis, mutations in codons 12 and 13 of *KRAS*, MSI phenotype, and *MGMT* methylation were assessed using techniques described previously [33], [36], [37]. Putative Lynch syndrome tumors were defined as tumors with one of the following characteristics: loss of immunohistochemical expression of MSH2 and MSH6, MSH6 alone, PMS2 alone, or MLH1 and PMS2 with wild-type *BRAF* and/or absence of *MLH1* methylation.

Mutations within exon 9 and exon 20 of the *PIK3CA* gene were assessed for each CRC case as follows. Briefly, 50–100 ng of formalin-fixed paraffin-embedded (FFPE) tumor DNA was amplified in a 15 µl PCR containing 7.5 µl of GoTaq master mix (Promega, Madison, WI, USA) and 5 pmoles of each primer (exon 9 forward 5′-GGG AAA AA TATG ACA AAG AAA GCT A-3′ and reverse 5′-TCC ATT TTA GCA CTT ACC TGT GAC-3′; exon 20 forward 5′-TTC TCA ATG ATG CTT GGC TCT GGA A-3′ and reverse 5′-AAT GCA TGC TGT TTA ATT GTG TGG A-3′). Reactions were cycled on a Biorad iCycler (Biorad, Hercules, CA, USA) using the following profile: 94°C for 2 minutes, followed by 45 cycles of 94°C for 15 seconds, 60°C for 15 seconds and 72°C for 15 seconds, cycling is finalized at 72°C for 5 minutes. PCR reactions were subsequently cleaned up using Montage PCR96 Cleanup plates (Millipore, Bedford, MA, USA) according to the manufacturer’s guidelines. Depending on the quality of the PCR product 1–4 µl was then used in an 8 µl sequencing reaction comprising 0.4 µl BigDye Terminator v3.1, 1.4 µl 5x reaction buffer and 1.5pmoles of primer (exon 9 5′-TAG CTA GAG ACA ATG AAT TAA GGG AAA-3′; exon 20 5′-TCT CAA TGA TGC TTG GCT CTG GAA-3′). Reactions were cycled for 96°C for 1 minute, followed by 25 cycles of 96°C for 10 seconds, 50°C for 5 seconds and 60°C for 90 seconds. Prior to running on an ABI3100 genetic analyzer (Applied Biosystems, Foster City, CA, USA), sequencing reactions were cleaned up using Xterminator reagent (Applied Biosystems, Foster City, CA, USA) according to the manufacturer’s instructions. Resultant sequences were analyzed using SeqMan software (DNASTAR, Madison, WI, USA).

### Statistical Analysis

Statistical analyses were performed with SPSS statistics software version 19.0 (SPSS Inc., Chicago, IL) for group comparisons and Stata version 11.1 (College Station, TX: StataCorp LP) for survival analysis. Comparisons for categorical variables were performed using Pearson’s chi-squared test or Fisher’s exact test where appropriate. Student’s *t*-test was used for comparisons of continuous variables.

Kaplan-Meier methods were used to estimate the survival by *PIK3CA* status (wild-type vs. mutated), with death from all causes as the endpoint and time measured from date of surgery until death or 31 December 2008, whichever came first. Survival was compared using the log-rank test. Cox regression models were used to estimate hazard ratios (HRs) associated with *PIK3CA* (wild-type vs. mutated), adjusting for: sex, age at diagnosis (<60, 60–70, >70 years), tumor location (proximal vs. distal), histologic grade (high vs. low), MSI status (MSI-high vs. non MSI-high), MGMT expression (normal vs. loss), *KRAS* and *BRAF* status (wild-type vs. mutated). This Cox regression was fitted for the whole cohort of patients and also after exclusion of patients with a *BRAF* mutated tumor. Results are presented as estimated HRs with 95% confidence intervals (CI) and Wald-test *P* values. The proportional hazards assumption was assessed using graphical methods and tests based on Schoenfeld residuals. A two-tailed *P* value was used for all analyses and values less than 0.05 were considered to be statistically significant.

## Results

### Clinical and Pathologic Characteristics of *PIK3CA*-mutated Colorectal Carcinoma

**Figure 1:**
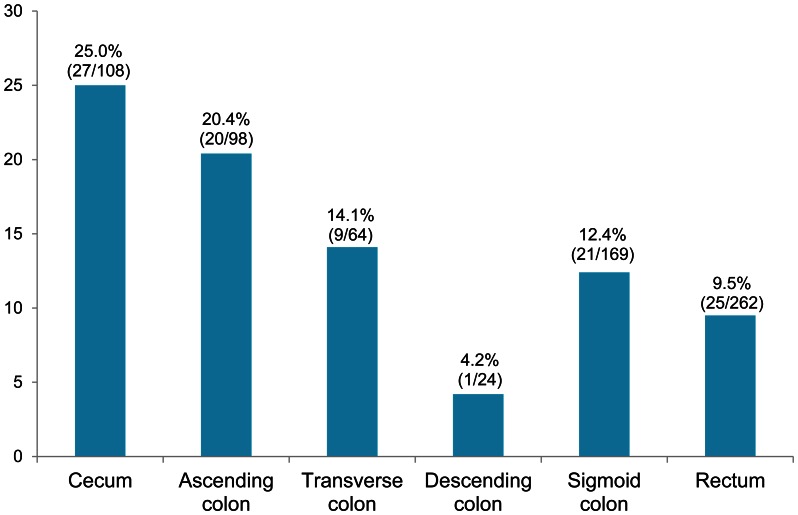
Proportion of *PIK3CA* mutation in colorectal carcinomas from each segment of the large bowel.

A complete set of clinical and molecular data was available for 757 patients. Characteristics of *PIK3CA*-mutated tumors are shown in **Table 2**. *PIK3CA* mutation was detected in 105 tumors (14%). The frequency of *PIK3CA* mutation did not differ significantly between males and females (*P* = 0.56), or between persons of Anglo-Celtic or southern European ethnicity (*P* = 0.22). No significant difference was found in age at diagnosis between patients with a *PIK3CA*-mutated carcinoma and those with wild-type *PIK3CA* carcinoma (*P* = 0.12). Compared with *PIK3CA* wild-type tumors, *PIK3CA*-mutated tumors were predominantly distributed in the proximal colon (54% vs. 34%, *P*<0.001) and were associated with focal or prominent mucinous differentiation (54% vs. 32%, *P*<0.001). **Figure 1** displays the frequencies of *PIK3CA* mutation in each segment of the large bowel, demonstrating a gradual decrease from the cecum to the rectum. No significant association was found with regard to tumor margin, histologic grade, lymph node status, or the presence of synchronous carcinoma or contiguous polyp (**Table 2**).

**Table 2:** *PIK3CA* mutation in 757 colorectal carcinomas (overall, exon 9 and exon 20 hot spots) and clinico-pathologic features.

Features	Classes	*PIK3CA* wild-type CRC	*PIK3CA* mutated CRC	*P* value*	Exon 9 Mutation	Exon 20 Mutation
Total		652	105		81	27
Mean age, years ± standard deviation		68±9	69±8	0.12	69±8	70±7
Male (%)		342 (53)	52 (50)	0.56	43/81 (53)	10/27 (37)
Anglo-Celtic Ethnicity (%)		443 (69)	78 (76)	0.22	58/78 (74)	23/27 (85)
Proximal colon location (%)		214 (34)	56 (54)	<0.001	***45/81 (56)***	***14/25 (56)***
High histologic grade (%)		147 (23)	21 (20)	0.51	19/80 (24)	2/26 (8)
Tumor margin (%)	Circumscribed	421 (72)	73 (76)	0.38	54 (74)	22 (85)
	Infiltrating	166 (28)	23 (24)		19 (26)	4 (15)
Mucinous differentiation (%)	Absent	402 (68)	43 (46)	<0.001	***32 (46)***	***11 (42)***
	Focal <50%	140 (24)	41 (44)		32 (46)	12 (46)
	Prominent >50%	46 (8)	9 (10)		6 (8)	3 (12)
Presence of TIL (%)		151 (24)	24 (24)	1.0	16/76 (21)	8/26 (31)
Presence of tumor budding (%)		203 (37)	33 (35)	0.75	28/74 (38)	5/23 (22)
Metastatic node (%)		251 (45)	37 (40)	0.38	31/72 (43)	7/24 (29)
Synchronous CRC (%)		13 (2)	3 (3)	0.59	3/79 (4)	0/25 (0)
Presence of contiguous polyp (%)		155 (25)	32 (32)	0.13	24/77 (31)	11/25 (44)

* *P* value comparing *PIK3CA* wild-type and *PIK3CA*-mutated tumors. There are 3 tumors with mutations in both exons 9 and 20. Results for exon 9 and exon 20 mutation in italic bold indicate statistical significance (*P*<0.05) when compared with *PIK3CA* wild-type tumors. CRC: Colorectal carcinoma; TIL: Tumor infiltrating lymphocytes.

### Associations of *PIK3CA* Mutation with Other Molecular Markers

**Figure 2:**
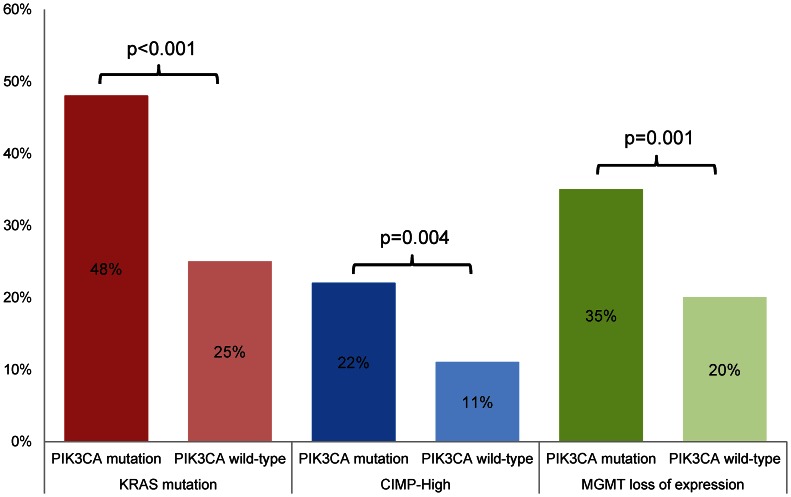
Associations between *PIK3CA* mutation and *KRAS* mutation, high levels of CpG island methylator phenotype (CIMP), and loss of MGMT expression.

We investigated associations between *PIK3CA* mutation and other molecular markers of colorectal carcinoma (**Table 3** **;** **Figure 2**). *KRAS* mutation was present in 50 of 105 (48%) *PIK3CA*-mutated tumors compared with 165 of 652 (25%) *PIK3CA* wild-type tumors (*P*<0.001). In addition, *PIK3CA*-mutated tumors were more likely to demonstrate high levels of CIMP (22%) when compared with *PIK3CA* wild-type tumors (11%) (*P* = 0.004). Though *BRAF* mutation was present in 14% of *PIK3CA*-mutated carcinomas, there was no significant difference in the prevalence of *BRAF* mutation between both tumor groups (*P* = 0.45). Decreased or loss of MGMT immunohistochemical expression was found more frequently in *PIK3CA*-mutated carcinomas compared with *PIK3CA* wild-type carcinomas (35% vs. 20%, *P* = 0.001). *MGMT* methylation (38% vs. 28%) and *MLH1* methylation (16% vs. 10%) were also more frequent in *PIK3CA*-mutated carcinomas compared with *PIK3CA* wild-type carcinomas, although neither reached statistical significance (*P* = 0.27 and *P* = 0.06, respectively). No significant association was found between the status of *PIK3CA* mutation and MSI status or the group of patients suspected of having Lynch syndrome based on their tumor molecular profile (putative Lynch syndrome; **Table 3**).

**Table 3:** *PIK3CA* mutation (overall, exon 9 and exon 20 hot spots) and other molecular characteristics of 757 colorectal carcinomas.

Features	*PIK3CA* wild-type CRC	*PIK3CA* mutated CRC	*P* value*	Exon 9 Mutation	Exon 20 Mutation
Total	652	105		81	27
*KRAS* mutation (%)	165 (25)	50 (48)	<0.001	***43/81 (53)***	10 (37)
*BRAF* mutation (%)	106 (17)	14 (14)	0.45	8/80 (10)	6/26 (23)
High levels of CIMP (%)	72 (11)	22 (22)	0.004	***16/77 (21)***	6/26 (23)
High levels of MSI (%)	75 (12)	14 (13)	0.58	6/80 (7)	***8/27 (30)***
*MLH1*methylation (%)	58 (10)	16 (16)	0.06	11/76 (14)	5/26 (19)
Putative Lynch syndrome^#^ (%)	36 (6)	6 (6)	0.93	2/80 (3)	4/27 (15)
*MGMT* methylation (%)	43 (28)	10 (38)	0.27	7/21 (33)	3/6 (50)
MGMT loss of expression (%)	122 (20)	33 (35)	0.001	***29/73 (40)***	5/25 (20)

* *P* value comparing *PIK3CA* wild-type and *PIK3CA*-mutated tumors. There are 3 tumors with mutations in both exons 9 and 20. ^#^Putative Lynch syndrome was defined as tumors with one of the following characteristics: loss of expression of MSH2 and MSH6, MSH6 alone, PMS2 alone, or loss of MLH1 and PMS2 expression with wild-type *BRAF* and/or absence of *MHL1* methylation. Results for exon 9 and exon 20 mutation in italic bold indicate statistical significance (*P*<0.05) when compared with *PIK3CA* wild-type tumors. CRC: Colorectal carcinoma; MSI: Microsatellite instability; CIMP: CpG island methylator phenotype.

### Specific Associations with Subtypes of *PIK3CA* Mutation

**Figure 3:**
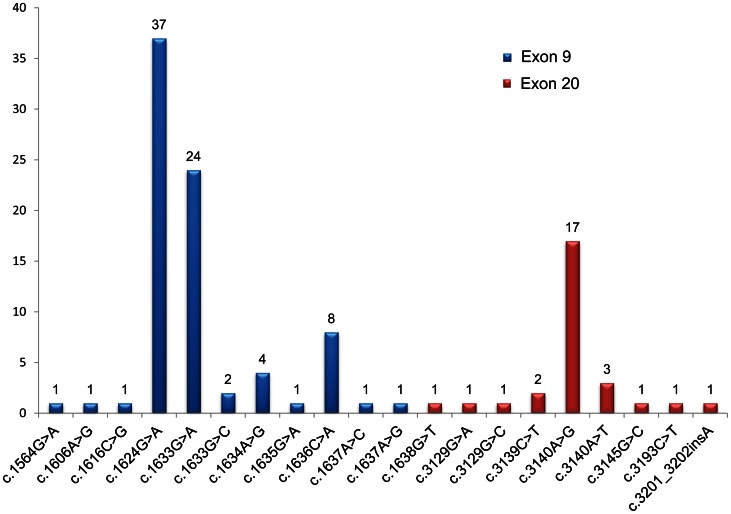
*PIK3CA* mutation subtypes.

Of all 105 tumors with *PIK3CA* mutations, 78 (74%) had a mutation within the helical domain of exon 9, with 1 tumor possessing 2 mutations, and 24 (23%) had a mutation in exon 20 catalytic domain. There were 3 tumors (3%) possessing mutations in both exon 9 and exon 20. The most common mutations were c.1624G>A (n = 37) and c.1633G>A (n = 24) in exon 9, and c.3140A>G (n = 17) in exon 20 (**Figure 3**). The most frequent *PIK3CA* mutation subtype was a G>A transversion in 63/105 (60%) of *PIK3CA*-mutated tumors. The G>A transversion was more frequently observed in tumors with loss of MGMT expression, compared with tumors showing normal MGMT expression (77% vs 50%; *P* = 0.048). No association was found between *MGMT* methylation and the G>A transversion mutation.

The significant associations between *PIK3CA* mutation and both proximal colon location and mucinous differentiation were found for each *PIK3CA* exonic mutation subgroup of tumors (**Table 2**). When compared with wild-type *PIK3CA* tumors, only exon 9 *PIK3CA* mutation was significantly associated with *KRAS* mutation (53% vs. 25%), high levels of CIMP (21% vs 11%) and loss of MGMT expression (40% vs. 20%), whereas these associations were not found or did not reach statistical significance for exon 20 *PIK3CA* mutation (37 vs. 25%, *P* = 0.17, 23% vs. 17%, *P* = 0.07 and 20% vs. 20% *P* = 0.99, respectively) (**Table 3**). On the other hand, only exon 20 *PIK3CA*-mutated carcinomas were more frequently MSI-high than *PIK3CA* wild-type carcinomas (30% vs. 12%; *P* = 0.005). Of the 14 tumors with both MSI-high and *PIK3CA* mutation, 8 possessed a mutation in exon 20. Of these 8 cases, 4 demonstrated *MLH1* methylation, and 4 were from patients with putative Lynch syndrome.

### Survival Analyses

**Figure 4:**
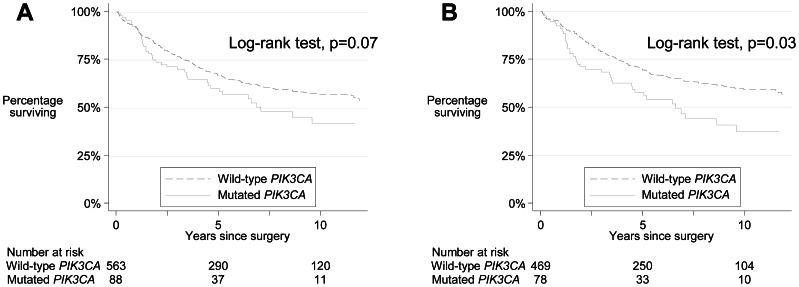
Kaplan-Meier survival curves showing overall survival according to *PIK3CA* mutation status (wild-type versus mutated) in (A) all colorectal carcinoma patients and (B) only in patients with *BRAF* wild-type tumor.

Of the 651 of the 757 patients (86%) with data available on all relevant variables, 261 died during a median follow-up of 5.03 years (range 5.1 months –16.7 years). There was some evidence of lower survival for those with *PIK3CA*-mutated tumors compared with wild-type *PIK3CA* tumors (log rank *P* = 0.07) (**Figure 4A**). After adjustment for other factors in a Cox regression model, this association was non-significant (HR 1.33, 95% CI 0.93–1.90, *P* = 0.12). After restricting the analysis to patients with a *BRAF* wild-type tumor, *PIK3CA* mutation was significantly associated with poorer overall survival (HR 1.51, 95% CI 1.04–2.19, *P* = 0.03) compared with wild-type *PIK3CA* (**Figure 4B**). The effect of *PIK3CA* on mortality was not significantly different according to *BRAF* status (*P*_interaction_ = 0.1). Although this interaction was not significant at the 5% level, such interaction analyses have low statistical power.

## Discussion

We detected *PIK3CA* mutation in 14% of colorectal carcinomas. These tumors were characterized by a predominant proximal colonic location, frequent presence of mucinous differentiation, and associations with *KRAS* mutation, high levels of CIMP and loss of MGMT immunohistochemical expression. Except for MGMT, the *P* values for these associations remained significant after the correction for multiple hypotheses testing was applied (20 variables compared between 2 groups; corrected *P* value = 0.05/40 = 0.00125). Though there was overlap with *BRAF* V600E mutation, the prevalence of *BRAF* V600E mutation did not differ between *PIK3CA*-mutated and *PIK3CA* wild-type tumors, suggesting that the association between *PIK3CA* mutation and high levels of CIMP is independent of *BRAF*.

Our estimate of the prevalence of *PIK3CA* mutation is in agreement with results from previous studies [4], [8], [9], [10], [11], [12], [13], [14], [15], [16], [17], [18], [19], [20], [21]. In this study, we used Sanger sequencing to identify *PIK3CA* mutations in DNA from FFPE tissue. When compared with pyrosequencing-based detection on FFPE DNA in a comparable population, the prevalence of *PIK3CA* mutation was slightly lower but not significantly different (14% vs. 16%; *P* = 0.14) [17], [29]. In agreement with Yamauchi et al, we found a gradual decrease in frequencies of *PIK3CA* mutation in tumors from the cecum (25%) to the rectum (9.5%), rather than an abrupt change from the proximal colon to the distal colon [38]. The most striking and consistent finding that we and others have found is the association between *PIK3CA* mutation and *KRAS* mutation in colorectal carcinoma, though some have not reported this finding [13], [28]. In our study, 47% of *PIK3CA*-mutated tumors also possessed *KRAS* mutations. Interestingly, the co-existence of *KRAS* mutation and *PIK3CA* mutation has also been found in other tumor types such as ovarian carcinoma [14], suggesting that such co-occurrence might provide a selective advantage to carcinoma cells from various origins.

The association between tumor mucinous differentiation and *PIK3CA* mutation has been previously reported [17], [30]. Other studies have shown that colorectal carcinomas with *KRAS* mutation display mucinous differentiation more frequently than carcinomas with wild-type *KRAS* [34], [39]. Given the strong association between *PIK3CA* mutation and *KRAS* mutation, this association is not unexpected. Likewise, loss of MGMT expression, another characteristic of *PIK3CA*-mutated carcinomas, has been reported to be more frequent in tumors with mucinous differentiation compared with tumors without mucinous differentiation [29]. Consistent with previous findings, MGMT loss of expression was associated with an increased frequency of G>A transversion mutations in *PIK3CA* [17].

Differences between tumors possessing exon 9 or exon 20 *PIK3CA* mutations have been reported by others [21], [29], [31], [40] but these reports should be interpreted with caution as the number of cases with mutations in exon 20 is low (25–30% of all *PIK3CA* mutations), increasing the likelihood of chance findings. We found that the associations between *PIK3CA* mutation and proximal colonic tumor location and mucinous differentiation were present in both exonic mutations, but the relationships with *KRAS* mutation, high levels of CIMP and MGMT loss of expression were confined to exon 9. It is possible that the absence of statistical significance between exon 20 *PIK3CA* mutation and *KRAS* mutation and CIMP might at least be partly caused by low sample size. The discrepancy between exons 9 and 20 mutations has not been universally reported with two previous large studies showing association with *KRAS* mutation regardless of exonic mutation in *PIK3CA* [21], [41] whereas Janku et al reported a significant association between *KRAS* mutation and exon 9 *PIK3CA* mutation [14]. A recent study of human p110-alpha in an avian model system has shown that activation induced by the helical domain in exon 9 requires RAS-GTP, whereas that induced by the kinase domain in exon 20 is functional in the absence of RAS-GTP and interacts directly with p85 [42]. Our findings of a stronger association between *PIK3CA* exon 9 mutation and activating mutations in codons 12 or 13 of *KRAS* supports this functional link between RAS signaling and activation of the helical domain of p110-alpha. A singular association was also observed between *PIK3CA* exon 20 mutation and MSI-high. This has been reported previously from 2 large studies [21], [29]. In this study, the MSI-high tumors with *PIK3CA* exon 20 mutations were equally distributed between those resulting from *MLH1* methylation (non-familial) and those likely to have resulted from a germline mismatch repair gene mutation (putative Lynch syndrome) suggesting no strong link between exon 20 mutations and either familial or non-familial MSI-high colorectal carcinomas, although the total number of tumors in this group was small (n = 8). Given the lack of association with the *BRAF* V600E mutation, as well as the fact that Lynch syndrome colorectal carcinomas arise from conventional adenomas, the association between exon 20 mutations and MSI-high might not be related to the serrated neoplasia pathway as has been previously suggested [21]. Furthermore, others have also described Lynch syndrome tumors having predominantly exon 20 mutations [40].

We found an association between *PIK3CA* mutation and poor overall survival for patients with a *BRAF* wild-type tumor. This result suggests that *PIK3CA* may have a mild adverse effect on survival after the strong effect of *BRAF* mutation is controlled [43]. Association between *PIK3CA* mutation and survival has been reported in previous studies with differences in the type of mutation or the group of patients: poor survival for all *PIK3CA* mutations in all colorectal carcinomas by Iida et al and Kato et al [13], [28], for tumors with both exons 9 and 20 mutations only by Liao et al [29], for tumors with exon 20 mutation only by Farina-Sarasquesta et al [32], and in patients with *KRAS* wild-type tumors only by Ogino et al [18]. Because of limited numbers we lacked power to demonstrate any difference in survival for the subset of patients with *PIK3CA* exon 20 mutation or patients with mutation in both exons 9 and 20. Recently, Liao et al reported that among patients with a *PIK3CA*-mutated colorectal cancer, regular use of aspirin was associated with significantly longer colorectal cancer–specific survival and overall survival, suggesting that aspirin use after diagnosis may have a differential effect on survival depending on *PIK3CA* mutation status [27]. It would be interesting to validate these findings in other series. Reliable data on aspirin use in our cohort of patients was not available to address this question.

In summary, we have found that *PIK3CA* mutation in colorectal carcinomas correlated with tumor proximal colonic location, mucinous differentiation, *KRAS* mutation, high levels CIMP and loss of MGMT expression. The adverse prognostic effect of *PIK3CA* mutation on survival was restricted to patients with a wild-type *BRAF* mutated tumor.
